# Ovarian survival after pelvic radiation: transposition until the age of 35 years

**DOI:** 10.1007/s00404-018-4883-5

**Published:** 2018-09-14

**Authors:** Ellen J. Hoekman, Dan Knoester, Alexander A. W. Peters, Frank W. Jansen, Cornelis D. de Kroon, Carina G. J. M. Hilders

**Affiliations:** 1 Department of gynecology, Leiden University Medical Center, P/O Box 9600, 2300RC Leiden, The Netherlands; 2 Department of Gynecology, Reinier de Graaf Gasthuis Delft, Delft, The Netherlands

**Keywords:** Ovarian preservation, Ovarian transposition, Radiation therapy

## Abstract

**Purpose:**

To evaluate the effectiveness of ovarian transposition (OT) prior to radiation therapy (RT) and to evaluate the effect of age on ovarian survival (OS) after OT.

**Methods:**

We performed a retrospective control study, with women (aged < 45 years) who underwent OT prior to pelvic radiation, versus women diagnosed with cervical cancer and treated with hysterectomy/trachelectomy and radiation therapy. All women were treated between 1989 and 2010. The 5 years OS rate was calculated, with a sub-analysis for age (25–30; 31–35 and 36–40 years). Ovarian failure was defined as climacteric complaints (with or without starting hormone replacement therapy) and/or laboratory measurements (FSH > 40 IU/L and/or estradiol < 100 pmol/L), or bilateral salpingo oophorectomy. Women were censored at recurrence.

**Results:**

Twenty-seven women after OT and 29 controls were included. The radiation dose was 44.8 Gy (25.0–63.0 Gy) and 46.3 Gy (45.0–50.0 Gy), respectively. The 5-year ovarian survival rate was 60.3% versus controls 0% (*p* < 0.001 95% CI 3.48–11.50). Despite the decrease in ovarian survival after OT with increasing age, in all age groups (25–30, 30–35 and 35–40) ovarian survival after OT was significantly better compared to women without OT (*p* = 0.001; *p* = 0.004 and *p* = 0.000, respectively). Neither intra-vaginal radiation therapy of concomitant chemotherapy in addition to pelvic radiation significantly altered ovarian survival.

**Conclusions:**

Our data shows that ovarian transposition prior to pelvic radiation is effective in women until the age of 35 years and needs to be discussed in patients aged 36–40 years.

## Introduction

Premature ovarian insufficiency (POI) after high gonadotoxic therapy is one of the side effects of cancer treatment with high impact on quality of life. Due to the continued refinements in treatment regimens, the rates of complete remission and cure have been improved. Thereby, quality of life after cancer is becoming a very important issue in a large number of women [1]. For example in the Netherlands, around 350 new cases of fertile patients are diagnosed with cervical cancer every year [2]. Furthermore besides cervical cancer, patients with Hodgkin’s disease, rectal cancer and other malignancies who need pelvic irradiation are at high risk where a small dose of irradiation can already cause infertility and POI [3, 4].

Infertility, climacteric symptoms such as vasomotor hot flashes, urogenital and sexual dysfunction and emotional disturbances are the side effects of POI with high impact on quality of life [5, 6]. Especially, estrogen withdrawal in young women is associated with decreased bone mineral density and impaired lipid profile with increased risk of ischemic heart disease [7, 8]. Although hormone replacement therapy is highly effective in counteracting these risks, long-term studies equating endogenous and exogenous estrogens are lacking [9]. Moreover, hormone replacement therapy (HRT) in women with POI may raise certain serious adverse side effects [10]. Finally, the success of this therapy depends largely on the patient’s compliance of the prescribed protocol [11]. As it has been shown, a small number of women follow their HRT regularly [12].

In an attempt to protect the ovaries from pelvic irradiation and prevent POI, ovaries can be transposed outside the radiation field, also known as ovarian transposition (OT). This surgical technique has been performed since 1952, with varying rates of success [13]. The last 30 years, POI after transposition ranges from 33 to 100%, using various techniques and modes of follow-up [14–28]. One reason of ovarian failure after OT may be the influence of scattered radiation therapy [29]. Moreover, it has been postulated that decreased blood flow to the ovaries, due to bending of the vessels as a result of the transposition, damage during surgery or as a side effect of the radiation therapy, increases the risk of POI. With regard to side effects of OT, the incidence of symptomatic ovarian cysts after OT is reported to range between 0 and 34% [15, 19, 22, 27]. In some of the reported cases, removal of the transposed ovary was necessary. More disturbingly, several studies have reported ovarian metastases in transposed ovaries [30, 31]. Thus, whether transposing the ovaries prior to radiation is a safe and effective procedure remains an ongoing issue.

We conducted a retrospective case–control study to evaluate the effectiveness and complications of ovarian transpositions prior to radiation therapy. Moreover, we evaluated the effect of age on ovarian survival after ovarian transposition.

## Materials and methods

From hospital databases of the Leiden University Medical Center, all patients were selected in whom ovarian transposition was performed between 1988 and 2010. From the same databases in which data were entered prospectively, women eligible for the control group were selected: pre-menopausal cervical cancer patients in whom the ovaries were not transposed prior to pelvic radiation.

### Data collection

Medical records of both cases and controls were reviewed for cancer treatment, survival, relapse and adjuvant cancer treatment. Since our study is retrospective by design, there was no obligation to have the protocol approved by our institutional review board.

With regard to ovarian survival, climacteric symptoms (irregular or no bleeding pattern, hot flushes), the use of hormonal replacement therapy and laboratory values (FSH and estradiol) were evaluated whenever available. Finally, complications of OT were assessed. Ovarian survival was defined as the absence of ovarian failure. Ovarian insufficiency was defined as follows: whenever at least two of the following criteria were reported, women were considered to suffer from ovarian failure: (1) climacteric symptoms, (2) use of hormonal substitution, (3) menopausal laboratory value (i.c. FSH > 40 IU/mL and/or estradiol < 100 pmol/L). Patients with normal ovarian function were censored at the date of the last follow-up with explicit data on the above-mentioned criteria for ovarian survival or at the date of systemic high gonadotoxic treatment for recurrence or metastatic disease. Follow-up of the ovarian function was continued until the first date of POI or in case of ovarian survival until a maximum of 5 years (when possible). Follow-up was reviewed until January 2018.

### Statistical analysis

The collected data was analyzed using SPPS 20^®^ (SPSS Inc, Chicago, IL, USA). Patient characteristics were compared with an independent *T* test. Overall ovarian survival was estimated by the Kaplan–Meier method, and the log-rank test was used to compare ovarian survival between cases and controls, the influence of age, additional chemotherapy and/or intra-vaginal radiation therapy. The Mann–Whitney *U* was performed to detect the difference of time until POI between OT and controls. With regard to age, the effect of RT after OT on ovarian survival was estimated for three different groups of age: 25–30 years, 31–35 years and 36–40 years. Figures were generated by Prism GraphPad 5 (GraphPad Software Inc., La Jolla, CA, USA).

## Results

The follow-up of 27 patients who underwent OT prior to pelvic radiation were reviewed (OT group). The majority of patients suffered from cervical squamous cell carcinoma. Other patients suffered from rectal adenocarcinoma (*n* = 3, stage 2, 3 and 3) or schwannoma (*n* = 1, grade II). Twenty-nine patients who suffered from cervical carcinoma and treated with pelvic radiation therapy (7 patients with adenocarcinoma, 22 patients with squamous cell carcinoma) in whom OT was not performed were included in the control group. In all women except one case (concomitant chemotherapy and pelvic radiation), surgery was the primary therapy. Characteristics, including details on adjuvant radiation therapy and concomitant chemotherapy of both cases and controls, are shown in Table 1.

**Table 1:** Patient characteristics

	Transposition	Control	*p* value
Mean age (years)	33.4	36.67	0.02
25–30	9	4	
31–35	7	10	
36–40	8	8	
40–44	3	7	
Primary cancer			
Cervical cancer	23	29	
Rectal cancer	3	0	
Schwannoma	1	0	
FIGO			
1b	15	25	
2a	6	3	
2b	2	1	
Other	4	0	
Lymph node status			NS
LN+	8	12	
LN−	19	17	
Post-operative therapy			
Mean external radiation dose (Gy)	44.88	46.32	NS
Mean internal radiation dose (Gy)	16.93	13.89	0.021
Chemoradiation treatment	9	0	
Adnex in situ			NS
1 adnexa	5	3	
2 adnexa	22	26	
Disease recurrence	5	5	
Diseased	3	5	

*LN* lymph node, *NS* non-significant

In most patients, standard follow-up treatment was performed according to the Dutch national guidelines for cancer treatment. The follow-up, for example of cervical cancer patients consists of a 3-month interval in the first 2 years, 6-month interval at year 3 and 4 and a 1-year interval at year 5. Patients were seen earlier on request or when there were complaints.

### Surgical procedure of OT

Ovarian transposition was performed during laparotomy (*n* = 18, 66.7%) for primary cancer treatment or by laparoscopy scheduled especially for OT (*n* = 9, 33.3%). In all surgical reports, the normal appearance of the ovaries was mentioned. The proprial ligament and fallopian tube were ligated and the ovarian vascularity in the infundibulopelvic ligament was dissected upward to allow transposition of the ovary to the upper abdomen above the level of the umbilicus. The ovary, along with the attached fallopian tube, was attached to the fascia of the anterior abdominal wall with a non-absorbable suture. No additional measurements were performed to reduce the risk of ovarian torsion. In 21 patients, the right ovary was transposed, while the left ovary was transposed in four patients and in two other patients both ovaries were transposed. In one patient, data about the transposition site were not known. The decision of transposing one ovary instead of two was due to uncertainty of additional risks such as the formation of cysts and torsion at the time.

### Additional cancer treatment

All patients received external pelvic radiation therapy with a mean dose of 44.8 Gy (range 25–63) in the OT group and 46.3 Gy (range 45–50) in the control group (NS). Ten patients of the OT group and 17 patients of the control group received additional brachytherapy (16.93 versus 13.89 Gy, *p* < 0.05) (Table 1). Nine patients received additional chemotherapy, mono or combined therapy of cisplatin, taxol and/or topotecan, respectively.

### Ovarian survival

The mean follow-up of OT patients, from RT until the latest date of normal ovarian function, was 34.5 months (min. 1.5–96.0).

In three cases, no details on ovarian function could be obtained for the medical records. These women were not included in the calculation of ovarian survival. One patient with cervical cancer did not experience menopausal complaints, despite elevated serum FSH (62 and 75 IU/mL) at two different measurements: although she strictly did not fulfill the criteria for ovarian failure, she was considered to be suffering from ovarian insufficiency from the date of the first elevated serum FSH.

**Fig. 1:**
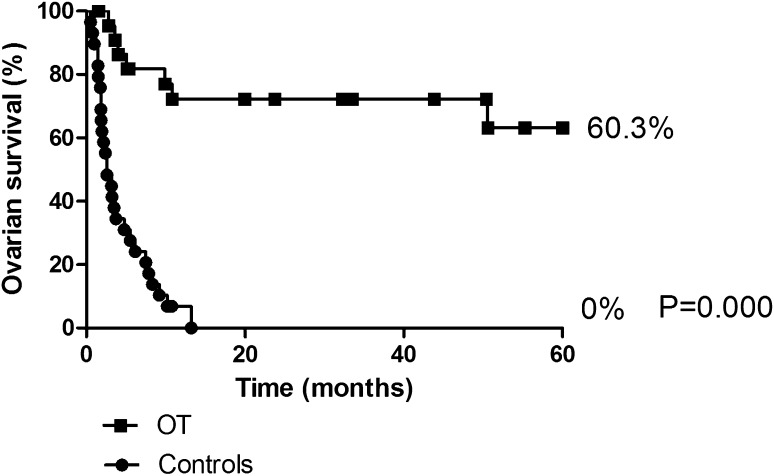
5 years ovarian survival, case and controls

Eight (29.6%) women in the OT group suffered from ovarian failure 5 years after pelvic radiation. Ovarian survival at 5 years was 60.3% (OT group) versus 0.0% (controls) (*p* < 0.001, (*p* < 0.000 95% CI 3.48–11.50) (Fig. 1). Median time to ovarian failure after radiation therapy was significantly shorter in the control group, 2.6 months, versus cases, 7.49 months (*p* = 0.009). Four out of the total of eight patients in whom OT failed suffered from ovarian failure within 6 months after radiation therapy. In two patients, the transposed ovaries (both ovarian survival at the time of surgery) were removed after, respectively, 2 (due to pseudomyxoma peritonei) and 45 months (preventive surgery because of MSH-6 mutation). These cases were censored for ovarian survival analyses at the date of surgery.

**Fig. 2:**
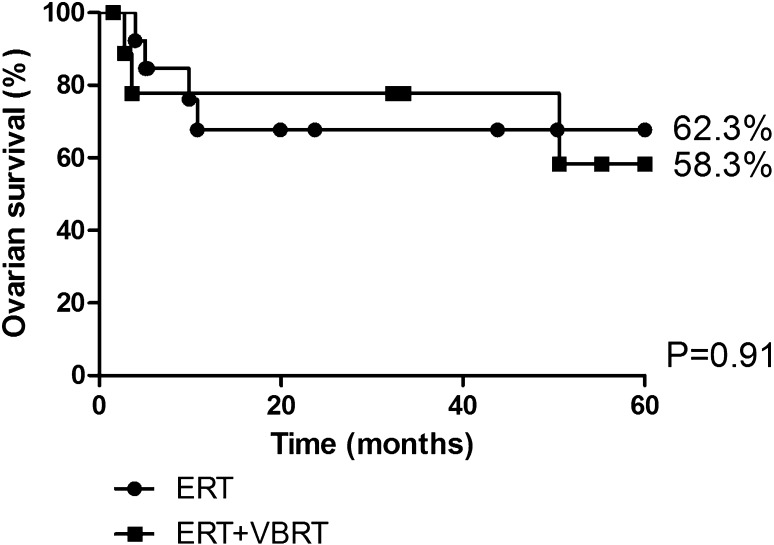
Ovarian survival after OT with or without intra-vaginal radiation therapy

Of the 24 patients in the OT group included in the ovarian survival analyses, 10 patients received external and intra-vaginal radiation therapy. Additional vaginal brachytherapy showed no significant difference in ovarian survival versus patients who received external radiation therapy alone: 5 years ovarian survival 58.3 versus 62.3% (*p* = 0.91, 95% CI 33.15–52.94, Fig. 2). Within the control group, the addition of vaginal brachytherapy did not show a significant difference in ovarian survival after 5 years either: the median time until ovarian failure after OT was 2.4 years without brachytherapy (95% CI 0.197–4.54) versus 2.6 years after the addition of brachytherapy (95% CI 1.24–3.96) (*p* = 0.501).

Nine patients received chemotherapy, of which two patients received chemotherapy after ovarian failure, one patient received 80 mg/kg cisplatin and was lost to follow-up, and another patient received taxol, ifosfamide and cisplatin and was censored at the start of the chemotherapy. The other five patients were included in the ovarian survival analyses and received concomitant pelvic radiation and chemotherapy (four patients received five or six doses of cisplatin 40 mg/m^2^ weekly schedule, and one patient received cisplatin 40 mg/m^2^ and topotecan weekly schedule). There was no significant difference in the ovarian survival of transposed ovaries receiving chemoradiation or radiation therapy alone, although the number of patients was low in this analysis (*p* = 0.41).

**Fig. 3:**
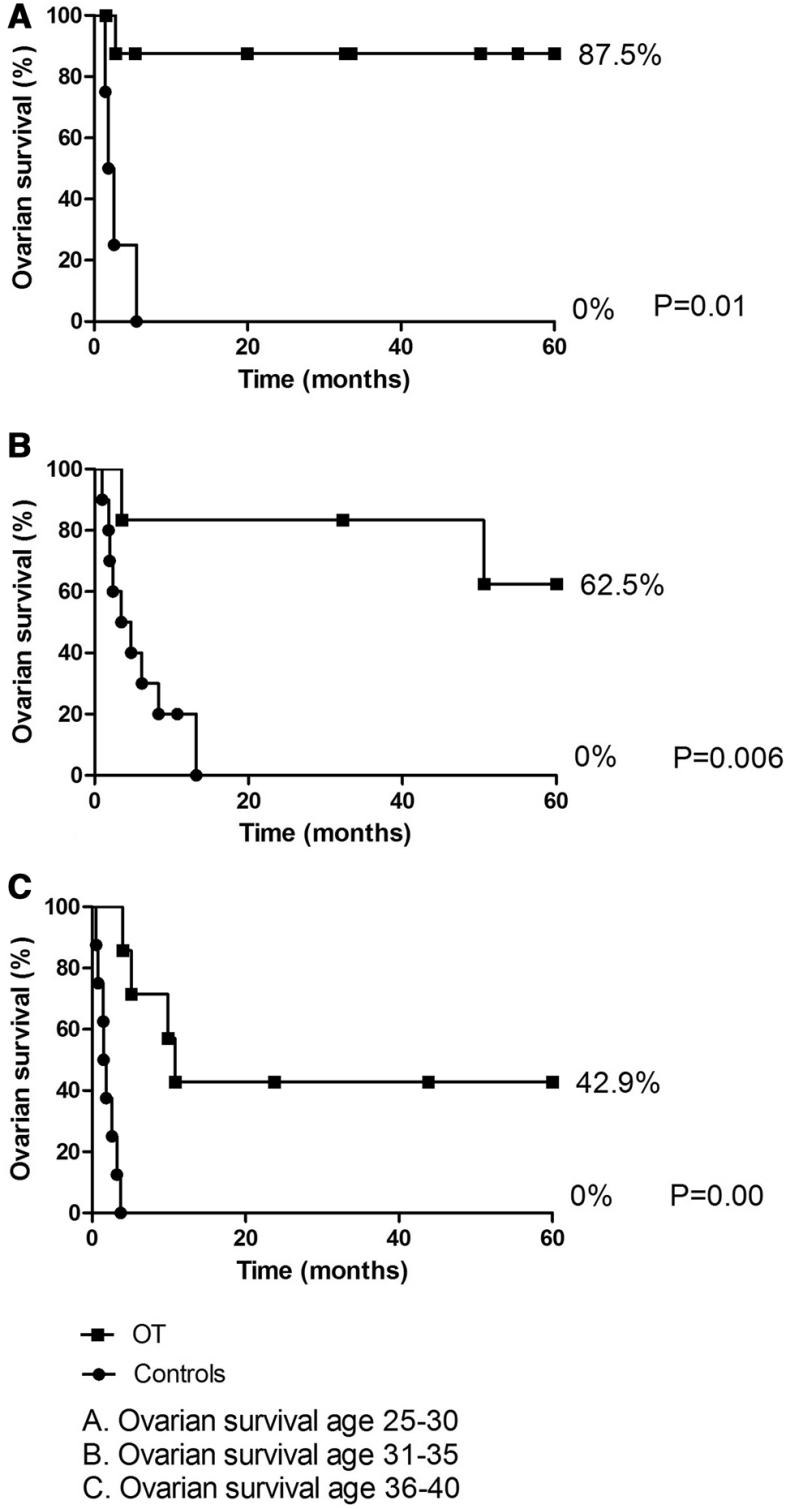
5-years ovarian survival after OT for different age groups

There was a non-significant decrease in 5 years ovarian survival after OT with increasing age (*p* = 0.16, Fig. 3). Despite the decrease in ovarian survival after OT with increasing age, in all age groups (25–30, 30–35 and 35–40) ovarian survival after OT was significantly better compared to women without OT (*p* = 0.001; *p* = 0.004 and *p* = 0.000, respectively (Fig. 3a–c). Although ovarian survival was significantly longer after OT in all the groups, when considering OT in women aged 35–40 years, we have to consider the small proportion of women aged 35–40 years who benefited from OT (two out of eight patients, Table 2).

**Table 2:** Patient characteristics of patients after ovarian transposition and aged 35–40 years

Nr.	Age at RT	Ovarian survival	Time (months)	Treatment	Notes
1	35.7	Yes	74.7	ERT	None
2	37.6	Yes	23.7	ERT	None
3	37.1	Yes	43.8	ERT	BSO
4	35.7	No	5.1	ERT	1 ovary in situ
5	35.8	No	10.8	ERT	Transposition of two ovaries
6	38.1	No	18.7	ERT	BSO
7	38.6	No	3.9	ERT	None
8	35.7	No	9.9	ERT and Cisplatin	None

In three patients, OT was performed at the age of 42 years: two of these patients were among the three women who were lost to follow-up and consequently not included in the survival analysis; the other 42-year-old woman had normal ovarian function 5 years after OT.

None of the patients had become pregnant during follow-up, and high technological surrogacy was not performed.

### Complications

All nine laparoscopic surgeries specially performed for ovarian transposition were uneventful. One ovarian torsion requiring oophorectomy of the transposed ovary (OT performed by laparotomy during primary surgery) was recorded. However, before oophorectomy, the patient was already diagnosed with POI despite OT. In none of the cases in this series, ovarian metastases were diagnosed during follow-up.

## Discussion

Our data show that ovarian transposition (OT) prior to pelvic radiation results in a significant increase in ovarian survival after pelvic radiation: 5 years ovarian survival 60.3% after OT versus 0.0% without OT (*p* < 0.001) Published rates of POI and complications after OT prior to pelvic radiation differ greatly (ovarian survival between 33% and 100%), which results in an ongoing debate about the safety and effectiveness of the procedure [14–28, 32]. However, most of the published data suffer from methodological flaws, since most studies are case series and non-comparative. Therefore, the results of effectiveness or disadvantages are not conclusive. The ovarian survival rates reported in this comparative study strongly support the advocates of ovarian transposition given its significant increase in ovarian survival after pelvic radiation and low complication rate.

Our results show that additional intra-vaginal radiation therapy and/or low dose chemotherapy (as part of chemoradiation therapy) has no significant impact on ovarian survival. Hence, these women should not be excluded from the option of OT. Unfortunately, due to small numbers it was not possible to compare patients after OT, receiving chemotherapy, EBRT and VBRT (*n* = 4), versus patients receiving EBRT and VBRT (without chemotherapy, *n* = 4). The PORTEC-2 trial showed the efficacy and reduced side effects of VBRT compared with external beam pelvic radiotherapy (EBRT) for patients with high–intermediate risk of endometrial cancer. Additionally, patients receiving VBRT only reported less bowel symptoms, which provided a better quality of life than after EBRT, contributing to the theory that VBRT delivers less scatter radiation that results in less impact on the surrounding tissue [33]. In contrast to our findings, Morice et all reported ovarian survival of 100% after OT, 90% after OT and brachytherapy and 60% after OT, brachytherapy and external radiation therapy [19]. Furthermore, ovarian survival after OT, external radiation therapy and brachytherapy has been described, respectively, in 33%; 80% and 100% [34–36]. Yamamoto reported a 5 year ovarian survival rate of 38.5% after OTs and radiation therapy [16]. Furthermore, Beukers reported ovarian survival in 41% of patients after OT and radiation therapy, with a mean follow-up of 43 months [26]. Hence there is no evidence to supports not transposing ovaries in case of pelvic chemoradiation in combination with intra-vaginal brachytherapy.

In the literature, the age above 40 years has often been used as a cutoff point. In our cohort three women older than 40 years underwent OT, of whom two were lost to follow-up. Hence, it is uncertain whether OT of women above the age of 40 years is worthwhile. Additionally, the ovarian survival was statistically significant in all age groups (Fig. 3), although on having a close look into the age group of 35–40 years only two out of eight patients (25%) experienced ovarian survival until 74 months (Table 2).

Previous studies have shown a correlation between age and the dose of pelvic radiation and ovarian failure. Hamish et al. described ovarian failure at the age of 10 years after exposure to 18.4 Gy, but at the age of 30 years ovarian failure occurred after exposure to 14.3 Gy [4]. The effect of OT in women above 40 years of age is limited, because of reduced fertilization and a higher risk of premature ovarian failure despite OT [19]. In addition, progressively smaller doses of chemotherapy are required to produce ovarian failure with increasing age [37–39]. In our opinion, to prevent POI, ovarian transposition should be discussed with all patients below the age of 35 years who need external radiation to the pelvis.

Despite OT, some women still suffer from ovarian failure after pelvic radiation. It has been shown that, due to scattered radiation, a substantial loss of ovarian function might still occur despite OT [21, 24, 40]. Furthermore, POI after OT and radiation therapy can be caused by migration of the ovaries back into the small pelvis after OT. Williams et al. reported ovarian failure which accounted in most patients of ovaries migrating back to the radiation area, as observed at repeat surgery [40]. An option for detecting the position of the ovaries is to attach a radio-opaque marker to the ovaries during transposition. The ovaries can be identified by imaging done for radiation planning and allows the radiotherapist to adjust the radiation field when necessary. Within our series, not all transposed ovaries were clipped by radio-opaque markers. Thus, in some cases it is unclear whether the ovary was still positioned outside the radiation field or had migrated back to the pelvis. In this context, we advise using radio-opaque markers to visualize the ovaries prior to and during radiation therapy. Finally, even in women after OT without radiation therapy, POI has been reported in up to 7% of women [15, 17, 18, 26]. Thus apart from damage due to radiation, multiple factors may contribute to failure of OT prior to radiation therapy.

Advocates of OT are supported by the data that POI has a high impact on the quality of life and may even cause more stress to the particular woman than suffering from cancer [25, 26]. Therefore, it is worthwhile to prevent POI and preserve ovarian function and/or fertility. However, complications due to OT should be weighted. The major adverse events related to OT are, however, rare: the occurrence of ovarian cysts, ovarian torsion and ovarian metastases on the transposed ovary [19, 20, 23]. In the presented series, adverse events were rare: an ovarian torsion was reported in the transposition group and was surgically removed. No ovarian metastases were reported in this study and surgical procedures were uneventful. In our opinion, OT is a safe procedure and should be offered to women below 40 years old whenever pelvic radiation is indicated.

In conclusion, our data show that ovarian transposition prior to pelvic radiation is effective, since both ovarian survival (*p* < 0.001) and time to POI (*p* < 0.01) are significantly better after OT prior to radiation therapy. Despite the low complication risk of ovarian transposition, we advise only ovarian transposition in women until the age of 35 years prior to pelvic radiation to prevent POI with its disadvantageous impact on the quality of life. In women aged 35–40 years, OT can be discussed when the procedure is executed during primary cancer surgery; however, the chances of long-term ovarian survival are limited.
